# Temporal and spatial heterogeneity of HER2 status in metastatic colorectal cancer

**DOI:** 10.1186/s13000-024-01508-y

**Published:** 2024-06-22

**Authors:** Flavia D’Angelo, Franck Monnien, Alexis Overs, Irvin Pem, Fanny Dor, Marine Abad, Sophie Felix, Zohair Selmani, Zaher Lakkis, Christophe Borg, Alexandre Doussot, Fréderic Bibeau, Chloé Molimard

**Affiliations:** 1 Department of Pathology, University Hospital of Besançon, 3 Boulevard Alexandre Fleming, Besancon, 25000 France; 2 Department of Oncobiology, University Hospital of Besançon, 3 Boulevard Alexandre Fleming, Besancon, 25000 France; 3 Department of Digestive Surgery, University Hospital of Besançon, 3 Boulevard Alexandre Fleming, Besancon, 25000 France; 4 Department of Oncology, University Hospital of Besançon, 3 Boulevard Alexandre Fleming, Besancon, 25000 France

**Keywords:** Colorectal cancer, HER2, Liver metastasis, Tumor heterogeneity

## Abstract

**Background:**

HER2-targeted therapies have recently emerged as an option in the management of metastatic colorectal cancer (mCRC) overexpressing HER2. However, data regarding HER2 status in primary CRC and its corresponding liver metastases are limited, potentially influencing clinical decisions. Therefore, the aim of this study was to compare the HER2 status in primary CRC and paired liver metastases.

**Methods:**

Patients with mCRC who were operated from their primary colorectal cancer and their corresponding synchronous or metachronous liver metastases, in the digestive surgery department of Besançon University Hospital, between April 1999 and October 2021, were included. Tissue microarrays were constructed from matched primary CRC and liver metastastic tissue samples. HER2 status was assessed by immunohistochemistry and in situ hybridization according to Valtorta’s criteria.

**Results:**

A series of 108 paired primary CRC and liver metastases, including a series of multiple liver metastases originating from the same patients (*n* = 24), were assessed. Among the primary CRC, 89 (82.4%), 17 (15.8%) and 2 (1.8%) cases were scored 0, 1 + and 2 + respectively. In liver metastases, 99 (91.7%), 7 (6.5%) and 2 (1.8%) were scored 0, 1 + and 2, respectively. Overall, there was a 19% discrepancy rate in HER2 status between primary CRC and metastases, which increased to 21% in cases with multiple synchronous or metachronous liver metastases in a given patient. No significant difference was found between metachronous and synchronous metastases regarding the HER2 status (*p* = 0.237).

**Conclusions:**

Our study highlights the temporal and spatial heterogeneity of HER2 status between primary CRC and corresponding liver metastases. These findings raise the question of a sequential evaluation of the HER2 status during disease progression, to provide the most suitable treatment strategy.

**Supplementary Information:**

The online version contains supplementary material available at 10.1186/s13000-024-01508-y.

## Background

Colorectal cancer (CRC) is the third most common cancer and the second leading cause of cancer related death worldwide with nearly 2 million new cases diagnosed and about 1 million death per year [1]. Almost 50% of CRC patients will develop liver metastases and less than a third will be candidates for surgical resection [2, 3].

The management of metastatic colorectal cancer (mCRC) depends on the resectability of the metastases, the patient’s condition and the tumor molecular features. In many cases, several biomarkers, such as *KRAS*, *NRAS*, *BRAF* and MisMatch Repair (MMR) status, are routinely assessed to adapt the therapeutic strategy [4]. Recently, the role of human epidermal growth factor 2 (HER2) as a new target has emerged in mCRC. HER2 is a strong oncogenic driver and trastuzumab, the first monoconal antibody blocking HER2, has become the standard treatment for HER2-positive advanced gastric cancer overexpressing HER2 [5, 6]. In mCRC, several phase II clinical trials have demonstrated the efficacy and tolerability of different dual HER2-targeted therapies [7–11]. However, this clinical efficacy was optimal in patients without *RAS* mutations [7]. More recently, a clinical trial evaluating trastuzumab conjugated to deruxtecan, a topoisomerase inhibitor, has shown promising activity in mCRC, irrespective of *RAS* mutation status [12, 13]. In these trials, patient recruitment is mainly based on immunohistochemistry and in situ hybridization. Indeed, in CRC, a specific HER2 scoring system, relying on these two techniques has been developed to provide an identification of CRC patients eligible in clinical trials [14, 15]. Moreover, *HER2* amplification has been associated to resistance to anti-EGFR treatment in wild-type *RAS* and *BRAF* mCRC.

In this setting, it is necessary to provide an accurate assessment of HER2 status. It can be challenging in cases where tumors show a heterogeneous expression of HER2 regarding different locations. Thus, in breast cancer and gastric cancer, it has been described that these situations can lead to discrepancies in HER2 status between primary tumors and metastases [16, 17]. In CRC, only few studies are available regarding HER2 heterogeneity. Moreover, most of them have been based on different scoring systems, with series including various number of cases [18–21]. In addition, spatial and temporal heterogeneity has never been precisely described [18–21].

Thus, the aim of this study was to compare the HER2 status between primary CRC and their corresponding liver metastases.

## Methods

### Patients

Patients who were operated for a primary CRC and underwent synchronous or metachronous liver metastases resection in the digestive surgery department of Besançon University Hospital, between April 1999 and October 2021, were selected for this study.

### Tissue microarray manufacturing

Tissue microarrays (TMA) were constructed from the most representative primary CRC and corresponding liver metastasis formalin-fixed paraffin embedded (FFPE) blocks. The punch's diameter was 1 mm and each tumor had three TMA spots. In addition, a supplementary TMA was built from the multiple synchronous or metachronous liver metastases present in the same patient.

### Determination of HER2 Status

#### HER2 Immunohistochemistry

HER2 immunohistochemistry (IHC) was initially assessed using 4 µm sections of TMA blocks. Immunostaining was performed on the Ventana Benchmark automatic immunostainer® (Roche diagnostics, Meylan, France), using a VENTANA anti-HER2/neu® (4B5) rabbit monoclonal primary antibody, according to the manufacturer’s instruction. In each section, there were external positive controls.

HER2 status of IHC staining was assessed according to Valtorta et al. [14]*.* It was defined as negative (0 no staining, 1 + faint staining regardless of cellularity, 2 + moderate staining with < 50% positive cells and 3 + intense staining with ≤ 10% positive cells), equivocal (2 + moderate staining with ≥ 50% of positive cells) and positive (3 + intense staining with > 10% positive cells) and scored by two pathologists. In cases of discrepancy, consensus was reached by reviewing cases where the pathologists’ interpretations initially differed.

#### Validation of TMA method for HER2 screening

To evaluate the reliability of the TMA method, an additional HER2 IHC on whole slides (WS) was performed for TMA spots with HER2 score of 1 + , 2 + , 3 + , as well as 10 randomly selected TMA spots IHC score of 0.

#### *HER2 fluorescent *in situ* hybridization*

Fluorescent in situ hybridization (FISH) was performed on WS CRC with an equivocal (2 + with ≥ 50% off positive cells) or positive (3 + with > 10% positive cells) HER2 IHC status. FISH using ZytoLight® SPEC ERBB2/CEN17 Dual Color Probe Kit (CliniSciences, Nanterre, France) according to the manufacturer’s instruction was used to assess *HER2* amplification. The scoring and evaluation were performed by counting ERBB2 and CEN17 signals from 100 non-overlapping nuclei core in tumor regions. Tumors with a ratio ERBB2/CEN17 ≥ 2 were considered amplified and otherwise were considered non-amplified [14].

### Patients’ characteristics

Clinical parameters were retrospectively collected by review of the medical files. These parameters included age, gender, WHO Performance Status at the diagnosis, neoadjuvant and/or adjuvant treatment, anatomical site and TNM stage according to UICC 8th edition.

The histological and molecular parameters collected included CRC histological type and grade according to the 2019 WHO Classification, lymphovascular and perineural invasion, lymph node status, MMR status and *KRAS*, *NRAS* and *BRAF* status.

### Statistical analysis

The HER2 IHC status in the primary tumor and corresponding liver metastases were expressed as percentages with 95% confidence interval (CI) and concordance was assessed using the Cohen’s kappa coefficient. The statistical analysis was performed with R software v.4.0.2.

### Ethics

The project was approved by the scientific board of the Regional Biobank of Franche-Comté, France (BB-0033–00024) ensuring patients’ informed consent. The study protocol conforms to the ethical guidelines of the 1975 Declaration of Helsinki (6th revision, 2008).

## Results

### Clinicopathological characteristics

**Fig. 1:**
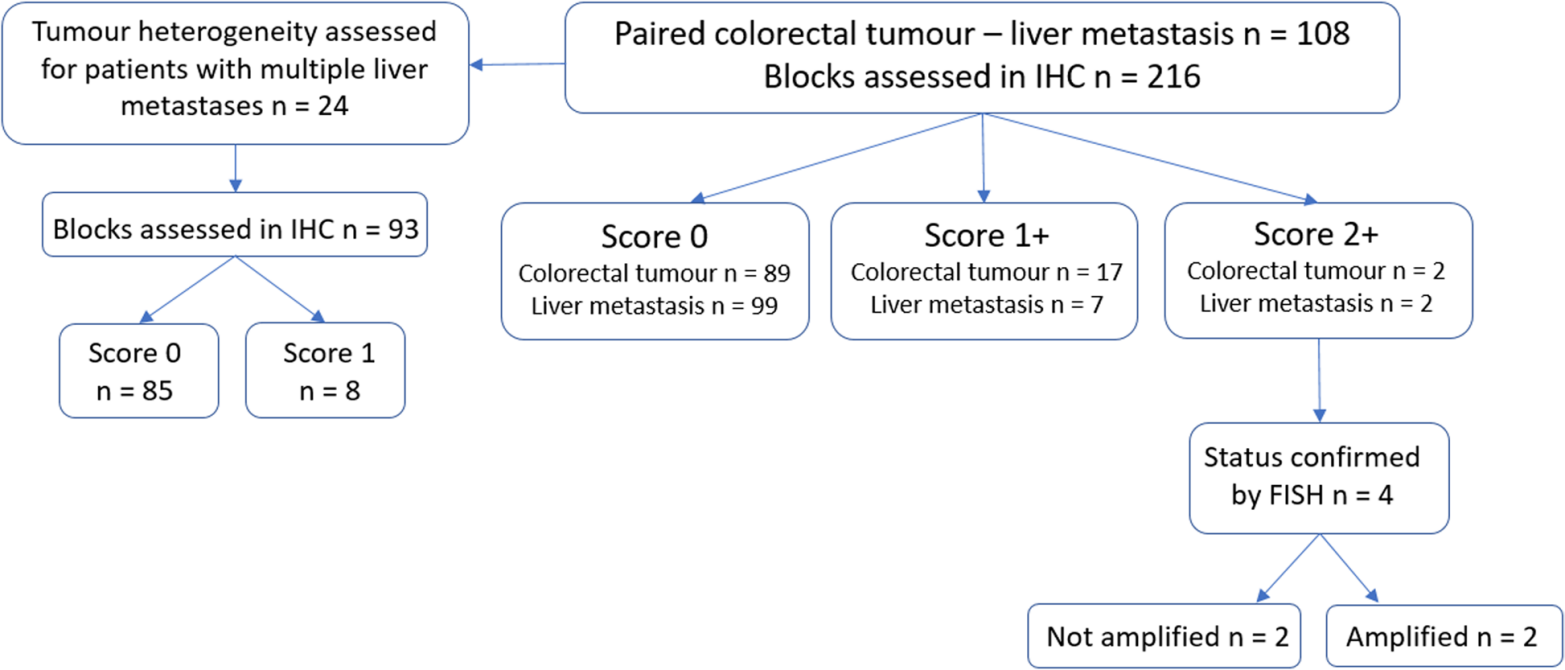
Flow chart. HER2 immunohistochemistry (IHC) was performed on the 108 paired colorectal tumor – liver metastasis. Two colorectal tumors and two liver metastases were scored 2 + and had their HER2 status assessed by fluorescent in situ hybridization (FISH). One pair (colorectal tumor and liver metastasis) was HER2 amplified and one pair was HER2 non-amplified. In addition, tumor heterogeneity was analyzed for 24 patients with multiple metastases. 85 metastases were scored 0 and 8 were scored 1 + on IHC

Tumor tissue samples from 108 patients who had colorectal and liver resection were collected (Fig. 1).

The relevant clinicopathological characteristics of the patients are summarized in Table 1.

**Table 1:** Clinicopathological characteristics of primary tumor from consecutive patients who underwent colorectal and liver metastasis resection for CRC

Characteristics	HER2 0 (*n* = 89)	HER2 1 + (*n* = 17)	HER2 2 + non amplified (*n* = 1)	HER2 2 + amplified (*n* = 1)
Age				
Mean	64.9	64.7	54	45
Sex				
Male	28	5	0	0
Female	61	12	1	1
Stage at diagnosis				
I	3	0	0	0
II	15	2	0	0
III	21	5	0	0
IV	50	10	1	1
Anatomic site				
Right-sided colon	21	5	1	0
Left-sided colon	29	8	0	1
Rectum	39	4	0	0
Histologic type				
NOS	86	16	1	1
Mucinous	3	1	0	0
Histologic grade				
Low grade	84	17	1	1
High grade	5	0	0	0
Lymphovascular invasion				
Present	54	9	1	1
Absent	35	8	0	0
Perineural invasion				
Present	31	3	1	1
Absent	58	14	0	0
Lymph node metastasis				
Present	60	11	1	0
Absent	29	6	0	1
Microsatellite status				
MSS	47	15	1	1
MSI	2	0	0	0
Unknown	40	2	0	0
*KRAS* status				
Mutated	31	9	1	0
Wild-type	32	5	0	0
Unknown	26	3	0	1
*NRAS* status				
Mutated	1	1	0	0
Wild-type	43	9	1	0
Unknown	45	7	0	1
*BRAF* status				
Mutated	1	0	0	0
Wild-type	57	14	1	0
Unknown	31	3	0	1
Neoadjuvant treatment				
Present	49	11	1	0
Absent	36	6	0	1
NA	4	0	0	0
Adjuvant treatment				
Present	67	11	1	1
Absent	15	5	0	0
NA	7	1	0	0
Liver metastases				
Synchronous	60	14	1	1
Metachronous	29	3	0	0

*NOS* not otherwise specified

*NA* not available

Seventy-six (70%) patients had synchronous liver metastases and 32 (30%) metachronous metastases.

### HER2 Status

The number of primary CRC with IHC scores of 0, 1 + and 2 + were 89 (82.4%), 17 (15.8%), and 2 (1.8%), respectively. The number of corresponding liver metastases with IHC scores of 0, 1 + and 2 + were 99 (91.7%), 7 (6.5%), and 2 (1.8%), respectively. None of the CRC was scored 3 + (Table 2).

**Table 2:** HER2 status in colorectal tumor and liver metastasis

	*HER2 status*					
	0	1 +	2 +	2 + /amplified	3 +	Total
Colorectal tumor	89	17	1	1	0	108
Liver metastasis	99	7	1	1	0	108
Total	188	24	2	2	0	

*IHC* immunohistochemistry,

0 no staining, 1 + faint staining, 2 + moderate staining, 3 + intense staining

A complete concordance between HER2 TMA and HER2 WS was observed in the 10 randomly selected patients with HER2 score 0.

**Fig. 2:**
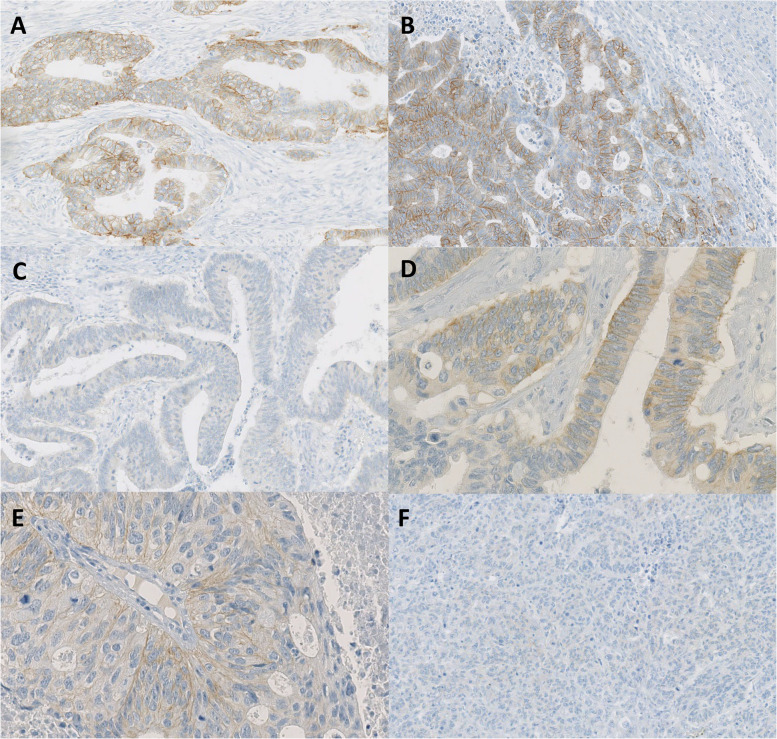
Illustration of anti-HER2 immunohistochemistry stain. Immunohistochemistry (IHC) score 2 + both on primary (**A**) and metastasis (**B**) in the HER2 amplified case (× 20). Example of IHC stain score 0 on the primary (× 20) (**C**) and on 1 + on the metastasis (× 40) (**D**). Example of IHC stain score 1 + on the primary (× 40) (**E**) and 0 on the metastasis (× 20) (**F**)

FISH detected *HER2* amplification in only one case (1/108; 0.9%) among the IHC 2+ samples, both present in the primary CRC and the corresponding liver metastasis (Fig 2).

This case corresponded to a 45 years old female patient having a low-grade NOS adenocarcinoma of the left side, associated with perforation and a synchronous liver metastasis, but without lymph node invasion. The patient was initially treated by surgery and adjuvant chemotherapy and progressed 3 years later with a pulmonary metastasis.

### Concordance of HER2 status between primary tumor and liver metastasis

The overall concordance between primary CRC and their paired liver metastasis was 80.5% (Table 3).

**Table 3:** HER2 status concordance between primary colorectal tumor and corresponding liver metastasis

*Liver HER2 status*		*Primary colorectal HER2 status*			
	0	1 +	2 +	2 + / amplified	Total
0	84	14	1	0	99
1 +	5	2	0	0	7
2 +	0	1	0	0	1
2 + / amplified	0	0	0	1	1
Total	89	17	1	1	108

0 no staining, 1 + faint staining, 2 + moderate staining

Out of 108 cases, 84 (77%), 2 (1.8%) and 1(0.92%) were respectively scored 0, 1 + , 2 + on both primary CRC and corresponding liver metastasis. For 21 patients (19%), the HER2 status of primary CRC was different from that on the liver metastasis. Five patients (4.6%) were scored 0 on primary CRC and 1 + on the liver metastasis (Fig. 2). Conversely, 14 patients (12%) showed 1 + staining on primary CRC and 0 on the liver metastasis (Fig. 2). One patient (0.92%) showed 1 + staining on primary CRC and 2 + on the liver metastasis and one patient (0.92%) showed 2 + staining on primary CRC and 0 on the liver metastasis. The Cohen’s kappa coefficient was 0.17 corresponding to a very low concordance.

In patients with concordant status, 28 (32.2%) had metachronous and 59 (67.8%) synchronous metastases. Among the 21 patients who presented a discrepancy in the HER2 status between the primary CRC and the metastasis, four (19.1%) had metachronous metastasis and 17 (80.9%) had synchronous metastasis. The characteristics of these patients with discordant HER2 status are summarized in the supplementary Table 1. A chi-square test was performed and showed no significant difference between metachronous and synchronous metastases regarding the HER2 status (*p* = 0.237).

### HER2 status in multiples liver metastases

**Fig. 3:**
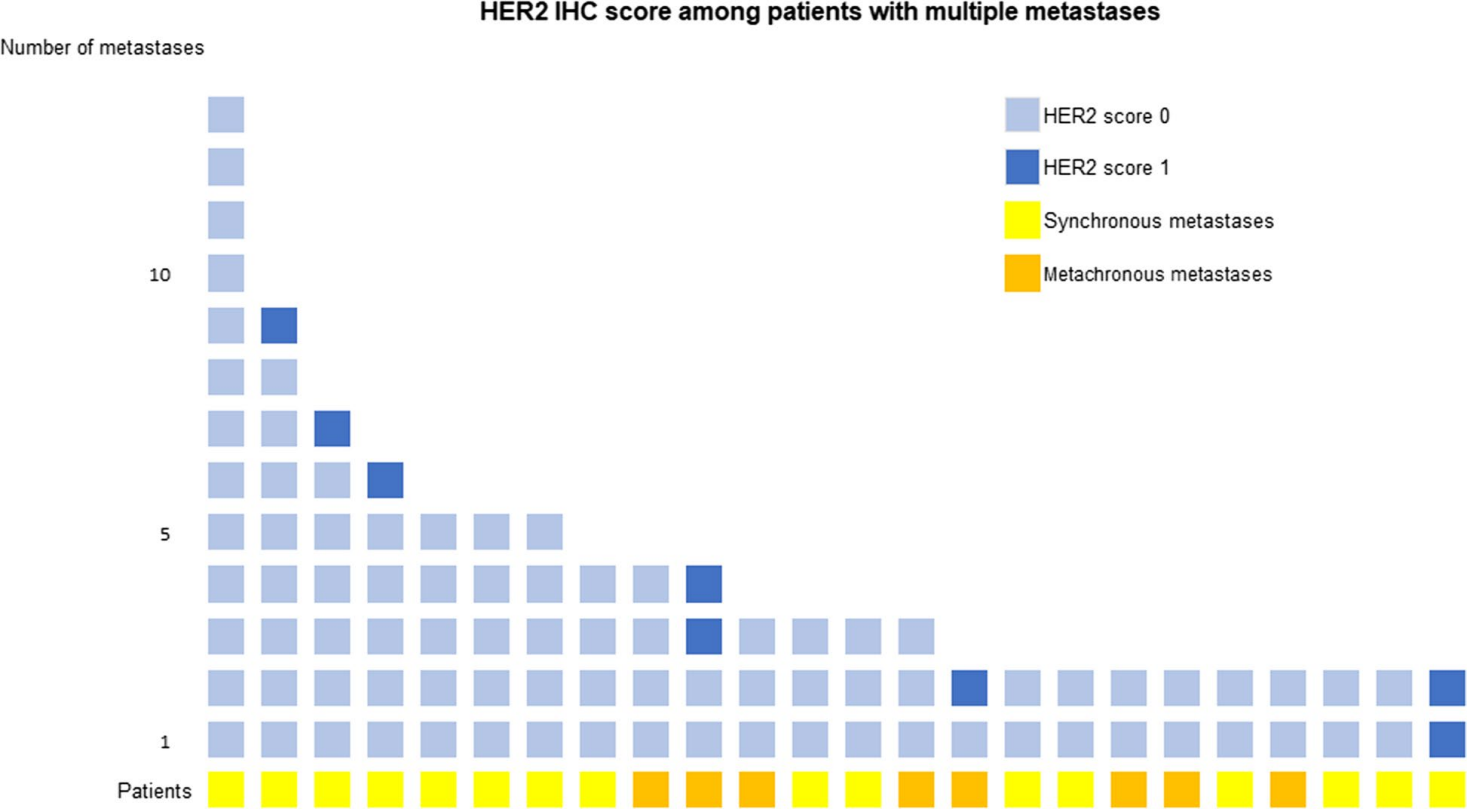
HER2 immunohistochemistry score among patients with multiple liver metastases*.* Each metastases of the 24 patients were represented according to their HER2 immunohistochemistry (IHC) score. Eight patients had metachronous metastases (orange) and 16 were diagnosed with synchronous metastases (yellow). Five patients showed a heterogeneous score

HER2 status was analyzed for 24 patients with multiple liver metastases. The number of metastases per patient varied from 2 to 13 lesions. Overall 8 (33.3%) were scored 1 + and 16 (66.7%) were scored 0. None of the metastases was scored 2 + or 3 + . For 5 out of 24 patients, liver metastases showed a different score, leading to a discrepancy reaching 21%. It concerned 2 patients with metachronous metastases and 3 patients with synchronous metastases (Fig. 3).

## Discussion

The aim of this study was to analyze the concordance of HER2 status between primary CRC and their corresponding liver metastases. Indeed, the precise evaluation of this biomarker is mandatory, as the expansion of new treatments targeting HER2 in this location has recently led to promising results, mainly in *RAS* wild-type tumors [7–13].

In our series, based on 108 patients and 285 samples, we found a significant discrepancy between primary CRC and its paired metastases reaching 19.5%. This rate reached 21% between the multiple liver metastases resected in each patient. This discrepancy concerned the 0, 1 + and 2 + IHC categories, as only one case of 2 + IHC *HER2* amplified CRC was observed, with the same status on primary and metastatic sites. This low frequency of *HER2* amplified CRC is in accordance with the literature’s data, reporting rates between 2 and 5% [22, 23].

Few studies have compared the HER2 status of primary CRC and its corresponding metastases [18–21, 24]. Moreover, they did not use the latest recommended scoring system, as compared to our work, based on the Valtorta criteria [14]. In addition, they did not analyze multiple synchronous or metachronous metastases originating from the same patient [18–21]. Lee et al. reported a discrepancy rate of 14.6% between primary CRC and liver metastasis. However, the interpretation of IHC staining was based on the criteria defined for gastric cancer [18]. In the study by Chen et al*.* discrepancy was also frequently observed in paired tumor samples encompassing primary CRC and brain metastases [24]. According to the study of Shan et al., a discrepancy in liver metastases compared to primary CRC was present in 27.3% of cases [20]. Recently, Hashimoto et al. found a discordance rate of 7% for HER2 amplified tumors and 19% for HER2 low tumors between primary CRC and metastases [21]. Additionally, we observed a discrepancy rate reaching 21% among the multiple liver metastases resected in a given patient. This rate was similar in synchronous and metachronous liver metastases. Thus, our work highlights the temporal and spatial heterogeneity of HER2 status that can be observed in CRC.

Our study took in consideration the “HER2 low status”, which includes 1 + and 2 + non-amplified cases, associated with a discrepancy rate reaching almost 19.5% between the primary CRC and its paired metastasis. This low level of HER2 expression represents an opportunity to offer a new approach with antibody–drug conjugate (ADC) such as trastuzumab deruxtecan (T-DXd) [12]. This therapeutic mechanism is supported by the ADC linking to HER2 protein found on malignant cells, even with low level of expression. After internalization and cleavage, DXd causes targeted DNA damage and apoptosis in cancer cells. Thus, it is a different pathway from the targeting of HER2 2 + amplified / HER2 3 + tumors, whose aim is to neutralize the oncogenic addiction provided by HER2 overexpression. This therapeutic approach of HER2 low tumors has been successfully validated in breast cancer, is promising in gastric cancer, but has not yet demonstrated positive effects in CRC. However, in this setting, only one study is available and clinical trials regarding this approach are still ongoing [12, 13, 25]. Therefore, this particular immunohistochemical pattern has still to be considered.

Theranostic biomarker heterogeneity remains a challenge in the management of solid tumors, potentially leading to under- or overtreatment. In this setting, many studies have been performed leading to different results according to the tumor type and the biomarker analyzed. Regarding the MMR status in CRC, the recent available studies demonstrated a high concordance rate between primary CRC and their metastases [26]. However, debate surrounds the *RAS* and *BRAF* status in primary CRC and corresponding metastases. While a review regarding multiple CRC biomarkers, including *RAS* and *BRAF* status*,* showed a strong agreement between the primary CRC and its metastatic site(s) [27], therapeutic pressure induced by chemotherapy and/or targeted treatment may alter the status post-treatment. The CRICKET study highlights how tumors initially *RAS* wild-type may become resistant to anti-EGFR therapy through the emergence of *RAS* mutated clones, and then recover a *RAS* wild-type status after stopping the targeted treatment [28]. These data illustrate dynamic tumor heterogeneity under treatment pressure.

Taken together, these data support the use of an approach that provides a more accurate assessment of the HER2 status and overcomes heterogeneity. In this setting, liquid biopsy relying on circulating tumor DNA (ctDNA), may offer a better way to characterize HER2 status in patients with metastatic CRC. Some clinical trials, such as the TRIUMPH study, have reported a very good concordance between liquid and tissue-based approaches [10]. However, this biomarker analysis was mainly designed to select HER2 amplified / 3 + tumors associated with a high level of DNA copy number, rather than to screen HER2 low tumors. As this assay is designed to detect DNA alterations, such as amplification in the blood, and not the absence or low level of protein expression represented by 0, and HER2 low CRC, which include 1 + and 2 + non amplified cases, the evaluation of HER2 by IHC remains relevant.

## Conclusions

In conclusion, our study highlights the temporal and spatial heterogeneity of HER2 status between the primary colorectal tumor and synchronous or metachronous liver metastases*.* Our data underline a difference between HER2 low CRC, which can be taken into account in this era of precision medicine and innovative therapeutic options, and raise the question of testing different tumor sites for HER2 status.

### Supplementary Information

Supplementary Material 1.

## Data Availability

No datasets were generated or analysed during the current study.

## Abbreviations

mCRC: Metastatic colorectal cancer
CRC: Colorectal cancer
MMR: MisMatch Repair
HER2: Human epidermal growth factor 2
TMA: Tissue microarrays
FFPE: Formalin-fixed paraffin embedded
IHC: Immunohistochemistry
FISH: Fluorescent in situ hybridization
WS: Whole slide
CI: Confidence interval
ADC: Antibody-drug conjugate
T-DXd: Trastuzumab deruxtecan
ctDNA: Circulating tumor DNA

## References

[1] Sung H, Ferlay J, Siegel RL, Laversanne M, Soerjomataram I, Jemal A (2021). Global cancer statistics 2020: GLOBOCAN estimates of incidence and mortality worldwide for 36 cancers in 185 countries. CA Cancer J Clin.

[2] Arnold M, Sierra MS, Laversanne M, Soerjomataram I, Jemal A, Bray F (2017). Global patterns and trends in colorectal cancer incidence and mortality. Gut.

[3] Imai K, Adam R, Baba H (2019). How to increase the resectability of initially unresectable colorectal liver metastases: A surgical perspective. Ann Gastroenterol Surgery.

[4] Cervantes A, Adam R, Roselló S, Arnold D, Normanno N, Taïeb J (2023). Metastatic colorectal cancer: ESMO clinical practice guideline for diagnosis, treatment and follow-up. Ann Oncol.

[5] Bang Y-J, Van Cutsem E, Feyereislova A, Chung HC, Shen L, Sawaki A (2010). Trastuzumab in combination with chemotherapy versus chemotherapy alone for treatment of HER2-positive advanced gastric or gastro-oesophageal junction cancer (ToGA): a phase 3, open-label, randomised controlled trial. Lancet.

[6] Alsina M, Arrazubi V, Diez M, Tabernero J (2023). Current developments in gastric cancer: from molecular profiling to treatment strategy. Nat Rev Gastroenterol Hepatol.

[7] Meric-Bernstam F, Hurwitz H, Raghav KPS, McWilliams RR, Fakih M, VanderWalde A (2019). Pertuzumab plus trastuzumab for HER2-amplified metastatic colorectal cancer (MyPathway): an updated report from a multicentre, open-label, phase 2a, multiple basket study. Lancet Oncol.

[8] Sartore-Bianchi A, Trusolino L, Martino C, Bencardino K, Lonardi S, Bergamo F (2016). Dual-targeted therapy with trastuzumab and lapatinib in treatment-refractory, KRAS codon 12/13 wild-type, HER2-positive metastatic colorectal cancer (HERACLES): a proof-of-concept, multicentre, open-label, phase 2 trial. Lancet Oncol.

[9] Gupta R, Meric-Bernstam F, Rothe M, Garrett-Mayer E, Mangat PK, D’Andre S (2022). Pertuzumab plus trastuzumab in patients with colorectal cancer with ERBB2 amplification or ERBB2/3 mutations: results from the TAPUR study. JCO Precis Oncol.

[10] Nakamura Y, Okamoto W, Sawada K, Komatsu Y, Kato K, Taniguchi H (2017). TRIUMPH Study: A multicenter Phase II study to evaluate efficacy and safety of combination therapy with trastuzumab and pertuzumab in patients with HER2-positive metastatic colorectal cancer (EPOC1602). Ann Oncol.

[11] Strickler JH, Cercek A, Siena S, André T, Ng K, Van Cutsem E (2023). Tucatinib plus trastuzumab for chemotherapy-refractory, HER2-positive, RAS wild-type unresectable or metastatic colorectal cancer (MOUNTAINEER): a multicentre, open-label, phase 2 study. Lancet Oncol.

[12] Siena S, Di Bartolomeo M, Raghav K, Masuishi T, Loupakis F, Kawakami H (2021). Trastuzumab deruxtecan (DS-8201) in patients with HER2-expressing metastatic colorectal cancer (DESTINY-CRC01): a multicentre, open-label, phase 2 trial. Lancet Oncol.

[13] Raghav KPS, Siena S, Takashima A, Kato T, Van Den Eynde M, Di Bartolomeo M (2023). Trastuzumab deruxtecan (T-DXd) in patients (pts) with HER2-overexpressing/amplified (HER2+) metastatic colorectal cancer (mCRC): Primary results from the multicenter, randomized, phase 2 DESTINY-CRC02 study. JCO.

[14] Valtorta E, Martino C, Sartore-Bianchi A, Penaullt-Llorca F, Viale G, Risio M (2015). Assessment of a HER2 scoring system for colorectal cancer: results from a validation study. Mod Pathol.

[15] Fujii S, Magliocco AM, Kim J, Okamoto W, Kim JE, Sawada K (2020). International harmonization of provisional diagnostic criteria for ERBB2-amplified metastatic colorectal cancer allowing for screening by next-generation sequencing panel. JCO Precis Oncol.

[16] Hanna WM, Rüschoff J, Bilous M, Coudry RA, Dowsett M, Osamura RY (2014). HER2 in situ hybridization in breast cancer: clinical implications of polysomy 17 and genetic heterogeneity. Mod Pathol.

[17] Creemers A, ter Veer E, de Waal L, Lodder P, Hooijer GKJ, van Grieken NCT (2017). Discordance in HER2 status in gastro-esophageal adenocarcinomas: a systematic review and meta-analysis. Sci Rep.

[18] Lee W-S, Park YH, Lee JN, Baek J-H, Lee T-H, Ha SY (2014). Comparison of HER2 expression between primary colorectal cancer and their corresponding metastases. Cancer Med.

[19] Styczen H, Nagelmeier I, Beissbarth T, Nietert M, Homayounfar K, Sprenger T (2015). HER-2 and HER-3 expression in liver metastases of patients with colorectal cancer. Oncotarget.

[20] Shan L, Lv Y, Bai B, Huang X, Zhu H (2018). Variability in HER2 expression between primary colorectal cancer and corresponding metastases. J Cancer Res Clin Oncol.

[21] Hashimoto T, Takayanagi D, Yonemaru J, Naka T, Nagashima K, Machida E (2023). A comprehensive appraisal of HER2 heterogeneity in HER2-amplified and HER2-low colorectal cancer. Br J Cancer.

[22] Siena S, Sartore-Bianchi A, Marsoni S, Hurwitz HI, McCall SJ, Penault-Llorca F (2018). Targeting the human epidermal growth factor receptor 2 (HER2) oncogene in colorectal cancer. Ann Oncol.

[23] Richman SD, Southward K, Chambers P, Cross D, Barrett J, Hemmings G (2016). HER2 overexpression and amplification as a potential therapeutic target in colorectal cancer: analysis of 3256 patients enrolled in the QUASAR, FOCUS and PICCOLO colorectal cancer trials. J Pathol.

[24] Chen P-C, Yeh Y-M, Chu C-T, Su P-F, Chiu P-H, Lin B-W (2023). HER2 amplification in colorectal cancer with brain metastasis: A propensity score matching study. Eur J Cancer.

[25] Babkoff A, Zick A, Hubert A, Tarantino P, Grinshpun A. Unleashing the power of anti-HER2 therapies in metastatic colorectal cancer: paving the way for a brighter future. ESMO Gastrointestinal Oncology. 2024;3. Available from: https://www.esmogastro.org/article/S2949-8198(23)00047-X/fulltext. Cited 2024 May 30. 10.1016/j.esmogo.2023.100032 PMC12836600 41648737

[26] Evrard C, Messina S, Sefrioui D, Frouin É, Auriault M-L, Chautard R (2022). Heterogeneity of mismatch repair status and microsatellite instability between primary tumour and metastasis and its implications for immunotherapy in colorectal cancers. Int J Mol Sci.

[27] Bhullar DS, Barriuso J, Mullamitha S, Saunders MP, O’Dwyer ST, Aziz O (2019). Biomarker concordance between primary colorectal cancer and its metastases. EBioMedicine.

[28] Cremolini C, Rossini D, Dell’Aquila E, Lonardi S, Conca E, Del Re M (2019). Rechallenge for patients with RAS and BRAF wild-type metastatic colorectal cancer with acquired resistance to first-line cetuximab and irinotecan. JAMA Oncol.

