# Supplementary material for: Temporal and spatial heterogeneity of HER2 status in metastatic colorectal cancer
Source: Diagn Pathol. 2024 Jun 22;19:88. doi: 10.1186/s13000-024-01508-y (PMC11193188; doi:10.1186/s13000-024-01508-y)
Supplement: Supplementary file 1 — Supplementary Material 1. [file 13000_2024_1508_MOESM1_ESM.docx]

**Supplementary Table 1.** Characteristics of patients with a discordant IHC status

|  | **IHC score primary / metastasis** | Localisation | Metastasis status | Histological type, grade | MMR status | RAS status | BRAF status | Neoadjuvant treatment | Adjuvant treatment |
| --- | --- | --- | --- | --- | --- | --- | --- | --- | --- |
| Patient n°1 | **2+/0** | Right colon | Synchronous | NOS, low-grade | pMMR | Mutated | Wt | Ct + bevacizumab | Ct + bevacizumab |
| Patient n°2 | **1+/0** | Right colon | Synchronous | NOS, low-grade | NA | Mutated | Wt | Ct + bevacizumab | Ct + bevacizumab |
| Patient n°3 | **1+/0** | Rectum | Synchronous | NOS, low-grade | pMMR | Mutated | Wt | Ct + bevacizumab | Ct |
| Patient n°4 | **1+/0** | Right colon | Synchronous | NOS, low-grade | pMMR | Wt | Wt | Ct + bevacizumab | Ct |
| Patient n°5 | **1+/0** | Left colon | Synchronous | NOS, low-grade | pMMR | Wt | Wt | Ct + bevacizumab | None |
| Patient n°6 | **1+/0** | Left colon | Synchronous | NOS, low-grade | pMMR | Mutated | Wt | None | Ct + bevacizumab |
| Patient n°7 | **1+/0** | Right colon | Metachronous | NOS, low-grade | pMMR | Mutated | Wt | None | None |
| Patient n°8 | **1+/0** | Right colon | Synchronous | NOS, low-grade | pMMR | Mutated | Wt | None | None |
| Patient n°9 | **1+/0** | Left colon | Synchronous | NOS, low-grade | pMMR | Wt | Wt | Ct | Ct |
| Patient n°10 | **1+/0** | Left colon | Synchronous | NOS, low-grade | pMMR | Mutated | Wt | Ct | Ct |
| Patient n°11 | **1+/0** | Rectum | Synchronous | NOS, low-grade | pMMR | Mutated | Wt | Ct + Rt | Ct |
| Patient n°12 | **1+/0** | Rectum | Synchronous | NOS, low-grade | pMMR | NA | NA | Ct | NA |
| Patient n°13 | **1+/0** | Right colon | Synchronous | Mucinous, low-grade | pMMR | Mutated | Wt | Ct | None |
| Patient n°14 | **1+/0** | Left colon | Metachronous | NOS, low-grade | pMMR | Mutated | Wt | None | Ct |
| Patient n°15 | **1+/0** | Rectum | Metachronous | NOS, low-grade | NA | NA | NA | None | None |
| Patient n°16 | **1+/2+** | Left colon | Synchronous | NOS, low-grade | pMMR | Mutated | Wt | None | Ct |
| Patient n°17 | **0/1+** | Rectum | Synchronous | NOS, low-grade | pMMR | Wt | Wt | Ct + Rt | Ct |
| Patient n°18 | **0/1+** | Left colon | Synchronous | NOS, low-grade | pMMR | Wt | Wt | None | Ct |
| Patient n°19 | **0/1+** | Rectum | Synchronous | NOS, low-grade | NA | Mutated | NA | Ct + Rt | Ct |
| Patient n°20 | **0/1+** | Left colon | Metachronous | NOS, low-grade | pMMR | Wt | Wt | None | Ct |
| Patient n°21 | **0/1+** | Right colon | Synchronous | NOS, low-grade | pMMR | NA | NA | None | Ct |

pMMR: proficient MisMatch Repair ; Wt: wild-type ; Ct: chemotherapy ; Rt: radiotherapy NA: not available
